# Letter from the Editor in Chief

**DOI:** 10.19102/icrm.2021.120807

**Published:** 2021-08-15

**Authors:** Moussa Mansour


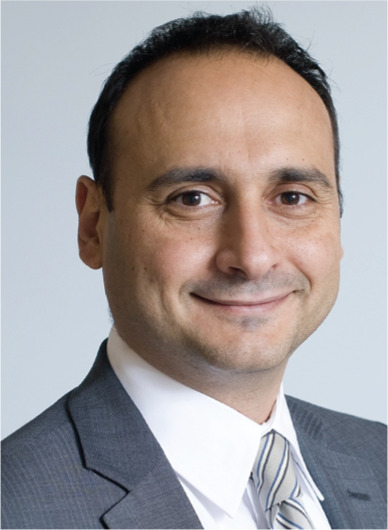


Dear Readers,

The annual scientific meeting of the Heart Rhythm Society took place recently in Boston as a hybrid event consisting of in-person and virtual attendance and presentations. High-quality science was presented, including outstanding late-breaking clinical trials sessions, which are my favorite part of this meeting.

Among the many important trials presented at the meeting, I found the Safety and Efficacy of Periprocedural Direct Oral Anticoagulant Versus Aspirin Use for Reduction of the Risk of Cerebrovascular Events in Patients Undergoing Ventricular Tachycardia (VT) Radiofrequency Catheter Ablation (STROKE-VT)^1^ to be particularly interesting. In this study, 246 patients were randomized to receive a direct oral anticoagulant (DOAC) or aspirin that was started within three hours after ablation. The patients underwent brain magnetic resonance imaging (MRI) within 24 hours of the procedure and again at 30 days after the procedure. Forty percent of the patients in each group had ischemic cardiomyopathy, and about 20% of the study participants underwent ablation for premature ventricular complexes rather than scar-related VT. One important procedural characteristic was access to the left ventricle, with more patients treated with a transseptal approach than a retrograde approach. The incidence of the primary endpoint of stroke was 0% in the DOAC group and 6.5% in the aspirin group, respectively. The brain MRI endpoint was also significantly different between the two groups: at 24 hours, MRI-detected asymptomatic events were noted in 12.2% of the DOAC group and 22.8% of the aspirin group, a difference that remained at 30 days (per the second MRI scan).

STROKE-VT is important for many reasons. It is the first study to investigate this topic, which has been studied extensively in the atrial fibrillation population but never before in the context of VT ablation. Second, it uncovered the high rate of cerebrovascular events that is associated with this type of ablation. Finally, it will likely result in a change in practice in favor of using DOACs in at least some groups of patients undergoing VT ablation. At the same time, however, some unanswered questions remain, including the feasibility of this approach in patients undergoing VT ablation in the presence of large-bore arterial hemodynamic support devices and patients undergoing extensive epicardial VT ablation, which can result in a higher risk of bleeding.

I hope that you enjoy reading this issue of *The Journal of Innovations in Cardiac Rhythm Management*.

Sincerely,


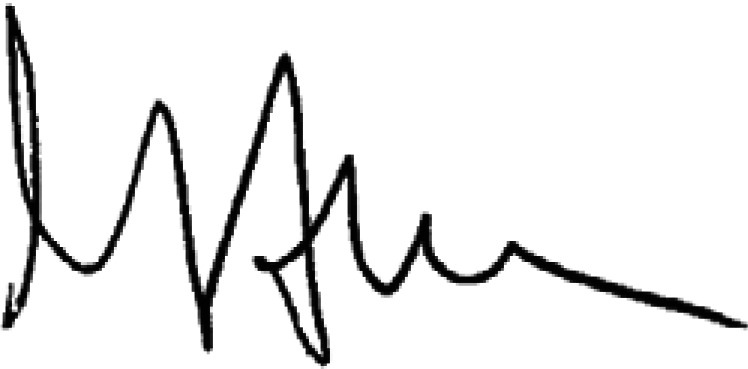


Moussa Mansour, md, fhrs, facc

Editor in Chief

*The Journal of Innovations in Cardiac Rhythm Management*

MMansour@InnovationsInCRM.com

Director, Atrial Fibrillation Program

Jeremy Ruskin and Dan Starks Endowed Chair in Cardiology

Massachusetts General Hospital

Boston, MA 02114
